# Variations in the pre-operative status of patients coming to primary hip replacement for osteoarthritis in European orthopaedic centres

**DOI:** 10.1186/1471-2474-10-19

**Published:** 2009-02-10

**Authors:** Paul Dieppe, Andrew Judge, Susan Williams, Ifeoma Ikwueke, Klaus-Peter Guenther, Markus Floeren, Joerg Huber, Thorvaldur Ingvarsson, Ian Learmonth, L Stefan Lohmander, Anna Nilsdotter, Wofhart Puhl, David Rowley, Robert Thieler, Karsten Dreinhoefer, EUROHIP Study Group

**Affiliations:** 1 Nuffield Department of Orthopaedic Surgery, University of Oxford, Nuffield Orthopaedic Centre, Windmill Road, Headington, Oxford, OX3 7LD, UK; 2 Department of Social Medicine, University of Bristol, Canynge Hall, Whiteladies Road, Bristol, UK; 3 Department of Orthopaedic Surgery, Carl-Gustav Carus University Hospital, Fetscherstr 74, D-01307 Dresden, Germany; 4 Department of Orthopaedics (RKU), University of Ulm, Oberer Eselsberg 45, D-89081 Ulm, Germany; 5 Department of Orthopaedic Surgery, Kantonsspital, CH-5001 Aarau, Switzerland; 6 Department of Orthopaedic Surgery, Akureyri University Hospital, 600 Akuyeri, Iceland; 7 Clinical Science at North Bristol, Medical School Unit, Southmead Hospital, Bristol, UK; 8 Department of Orthopaedics, Clinical Sciences, [SL] Lund University, Lund University Hospital, SE-22185 Lund, Sweden; 9 Department of Orthopaedics, Halmstad Central Hospital, S- 301 85 Halmstad, Sweden; 10 Department of Orthopaedic & Trauma Surgery, Ninewells Hospital and Medical School, Tayside Orthopaedic Rehabilitation Technology Centre, Dundee, UK; 11 Department of Rheumatology and Rehabilitation, Stadtspital Triemli, Birmensdorferstr 497, CH-8063 Zurich, Switzerland

## Abstract

**Background:**

Total hip joint replacement (THR) is a high volume, effective intervention for hip osteoarthritis (OA). However, indications and determinants of outcome remain unclear. The 'EUROHIP consortium' has undertaken a cohort study to investigate these questions. This paper describes the variations in disease severity in this cohort and the relationships between clinical and radiographic severity, and explores some of the determinants of variation.

**Methods:**

A minimum of 50 consecutive, consenting patients coming to primary THR for primary hip OA in each of the 20 participating orthopaedic centres entered the study. Pre-operative data included demographics, employment and educational attainment, drug utilisation, and involvement of other joints. Each subject completed the Western Ontario and McMaster Universities Osteoarthritis Index (WOMAC – Likert version 3.1). Other data collected at the time of surgery included the prosthesis used and American Society of Anaesthesiologists (ASA) status. Pre-operative radiographs were read by the same three readers for Kellgren and Lawrence (K&L) grading and Osteoarthritis Research Society International (OARSI) atlas features. Regression analyses were carried out.

**Results:**

Data from 1327 subjects has been analysed. The mean age of the group was 65.7 years, and there were more women (53.4%) than men. Most (79%) were ASA status 1 or 2. Reported disease duration was 5 years or less in 69.2%. Disease in other joint sites was common.

Radiographs were available in 1051 subjects and the K&L grade was 3 or 4 in 95.8%. There was much more variation in clinical severity (WOMAC score); the mean total WOMAC score was 59.2 (SD 16.1). The radiographic severity showed no correlation with WOMAC scores.

Significantly higher WOMAC scores (worse disease) were seen in older people, women, those with obesity, those with worse general health, and those with lower educational attainment.

**Conclusion:**

1. Clinical disease severity varies widely at the time of THR for OA.

2. In advanced hip OA clinical severity shows no correlation with radiographic severity.

3. Simple scores of pain and disability do not reflect the complexity of decision-making about who should have a THR.

## Background

Total hip replacement (THR) is an effective and cost-effective procedure for people with severe hip osteoarthritis (OA), unresponsive to conservative therapy [1,2]. It has become a high volume procedure throughout Europe, and annual rates continue to rise [2,3].

It is generally accepted that the indications for THR are pain and disability in spite of the use of non-surgical interventions such as education, drugs, walking aids and physical therapy [4]. However, it is not clear how severe the pain or disability needs to be before surgery should be undertaken, or when in the course of OA it is most appropriate to perform a THR. Economic modelling [5], some outcome studies [6] and patient views [7] suggest that it might be appropriate to perform surgery early on, before the condition gets too severe. However, doctors tend to be cautious about the use of a potentially dangerous and irreversible intervention too early, particularly because of evidence that it is not universally successful [8] and the accumulating risk with time of late prosthesis loosening necessitating further, more complex surgery. The potential implications of operating relatively early or late in the course of the disease are large, as this could have massive effects on the volume of surgery undertaken, as well as on outcomes.

There have been a number of attempts to develop indications for THR through consensus procedures [9], appropriateness criteria [10], and criteria for the prioritisation of patients waiting for a THR [11,12]. However, the resulting publications have done little more than emphasise the importance of pain and physical disability, and they struggle to take account of other factors that affect surgical decision making, such as co-morbidities, individual values and aspirations, and psycho-social circumstances. Furthermore, they do not address the issues of relative risk of complications or of a poor outcome, or how these might be affected by variations in THR indications.

The 'EUROHIP' consortium has been addressing the current debate on the utilisation and timing of THR, by studying the indications for primary hip replacement [13,14], and the process of hip replacement in the different centres involved [15]. In addition, the group has developed a cohort of people undergoing primary hip replacement for primary OA. The overall aims of the 'EUROHIP' cohort study are threefold: 1) to describe the amount of variation in disease severity at the time of primary THR in Europe; 2) to look for determinants of any variation; 3) to examine the effects of variations and their determinants on short-term (one year) patient centred outcomes. In this paper we report on the first two of these aims – describing the cohort and the variations in disease severity observed at the baseline assessment, and exploring some of the determinants of variations in pre-operative disease severity.

## Methods

### Participating centres and patients

The 'EUROHIP' consortium includes 20 orthopaedic centres in 12 different European countries. The overall design was endorsed by representatives from each centre in 2002. It was agreed that we would recruit a large multi-centre cohort of patients undergoing primary THR willing and able to complete self-administered questionnaires recording demographic variables and pre-operative levels of pain, stiffness, mobility and quality of life (using standard, validated questionnaires) as well as their expectations of the operation. One year after surgery they would be sent a similar questionnaire in the post. In addition, we agreed to obtain pre-operative radiographs and to record data about the operative procedure, including the type of prosthesis used. Inclusion criteria included a diagnosis of OA of the hip, primary hip replacement, and signed informed consent; exclusion criteria included causes of hip disease other than OA, severe mental illness or dementia, and patients unwilling or unable to take part in the study.

### Data collection

Each centre obtained local ethical approval, if required. The study protocol and data collection forms were designed in Bristol, UK and Ulm, Germany by the study PIs (PAD and KD) and the study co-ordinator (SW). The patient questionnaire was piloted for acceptability in Bristol and modified accordingly before being sent to Ulm for translation and distribution. Questionnaires were sent to each centre for translation and returned for checking before printing and distribution with a set of instructions. All data forms included the birth year and initials of the patient, as well as a centre ID and an individual patient number, to ensure unique identification while maintaining patient anonymity. Full identification records of all patients were kept separately in each centre. Completed questionnaires were photocopied locally and then returned to Bristol, where the database was constructed.

Prior to surgery, patients completed a questionnaire about age, sex, home circumstances, employment, education, current medications, duration of pain in the hip to be replaced and problems in other joints. In addition, they completed the WOMAC [16] and EQ5D [17] questionnaires (the EQ5D data has not been included in any of the analyses presented below).

### The Western Ontario and McMaster Universities (WOMAC) OA Index

The WOMAC index (version 3.1) was used to assess the severity of symptoms. This consists of 24 items in 3 subscales: pain (5 items), stiffness (2), and physical function (17) [16]. Component items are measured on a 5-point Likert scale with higher scores indicating greater symptom severity (0 = none, 1 = slight, 2 = moderate, 3 = severe, and 4 = extreme). Missing data were treated as follows: if ≥ 2 pain, both stiffness, or ≥ 4 physical function items were not completed, the items were regarded as invalid, and the subscale score not calculated; where 1 pain, 1 stiffness, or 1–3 physical function items were missing, the average value for the subscale was used in place of the missing item. A total score was calculated for each subscale, and a normalised score (0 indicating no symptoms and 100 indicating extreme symptoms) then calculated for each subscale, by summing up the total score of each subscale, multiplying it by 100, and dividing by the possible maximum score for the scale. A total score out of 96 was created by combining the 3 subscales. This was then converted into a normalised score out of 100, as described in the WOMAC user's handbook.

The surgical teams were asked to complete a form recording the patient's height and weight (from which BMI was calculated), side of surgery, duration of arthritis, date wait-listed and date of surgery. The form also asked for prosthesis type, ASA status – a standard measure of fitness for surgery, scored in this study from 1 (normal, healthy) to 4 (life-threatening systemic disease) [18].

### The Kellgren and Lawrence (K&L) Radiographic scores

The K&L score was used to assess structural disease severity. A pre-operative anterior posterior (AP) radiograph of the pelvis was obtained from all patients within 6 months of surgery. In order to standardise readings all films were examined by one of three observers within the co-ordinating centre (Bristol) who undertook training sessions together. These three observers all read 20 randomly selected films on two occasions, in a random order, to test their inter- and intra-rater reliability using Kappa scores. They collected data on the hip to be operated on, including the standard K&L grade (0–4) [19] and the intra-articular pattern of disease distribution (supero-lateral, supero-medial, medial or concentric) and whether the hip disease appeared hypertrophic (excessive osteophytes and new bone formation) or atrophic (extensive bone loss).

As most of the radiographs showed advanced OA, we divided K&L grades 3 and 4 further by adding data from the individual scores of joint space narrowing and bone attrition (assessed using the OARSI atlas – [20]). A K&L grade 3 radiograph with joint space narrowing (grade 1 on the OARSI atlas) was graded 3a, those with more severe joint space narrowing (OARSI atlas grade 2) 3b. Similarly, a K&L grade 4 radiograph (which has complete loss of joint space, graded 3 on the OARSI atlas) was divided into 4a if there was no bone attrition seen, either in the femur or acetabulum, and 4b if there was any bone attrition noted in any part of the joint.

### Database management and statistical analysis

Each centre was given 18 months to collect data. A 'Microsoft Access' database was set up in Bristol where all patient data was entered and checked by trained staff. In 2006, when the study had almost been completed and the database was about to be closed, the analysis plan was agreed and some remaining missing data obtained from participants. The database was then closed and a professional database manager carried out routine data cleaning.

Stata 9.2 was used for all statistical analyses (Stata Corp., College Station, TX). The main outcomes in the analysis were the three WOMAC subscale scores (pain, stiffness, function) and the combined total WOMAC score. Exposure variables considered in analyses were: Age (< 50, 50–69, 70+), Gender, Obesity (not obese [BMI < 30], obese [BMI 30–39], morbidly obese [BMI > 40]), Employment status (employed, retired, retired early, other), Education after leaving school (none, diploma or equivalent, degree, postgraduate degree), ASA status (1, 2, 3, 4), and Kellgren & Lawrence grade (0, 1, 2, 3, 4) of the hip operated on.

Univariate linear regression analysis was performed to explore the association between the outcome with each exposure, and a multivariate regression analysis then carried out to control for confounders. The distribution of WOMAC scores was assessed to examine the assumption of normality. Wald tests were used to explore linear trends, by fitting models with the variable as a score. To assess for non-linear trends, likelihood ratio tests were used, comparing a model with a categorical variable to that with the variable as a score. Effect modification was considered using likelihood ratio tests to examine for interaction between age, sex and obesity.

## Results

### 1. The cohort and demography of patients included in the analysis

A total of 1520 patients were entered from the 20 centres, an average of 76 patients/centre (range 41–167) (Table 1).

**Table 1:** EUROHIP Participating orthopaedic centres of excellence, and the number of patients entered into the cohort study reported here.

Country	City	Patients entered
Austria	Innsbruck	119
Austria	Vienna	44
Finland	Helsinki	48
France	Paris	46
Germany	Dresden	160
Germany	Hamburg	167
Germany	Heidelberg	47
Germany	Magdeburg	49
Germany	Ulm	152
Hungary	Szeged	70
Iceland	Akyreyri	79
Italy	Turin	66
Poland	Warsaw	50
Spain	Madrid	53
Sweden	Helsingborg	41
Sweden	Karlshamm	70
Switzerland	Aarau	52
Switzerland	Zurich	60
UK	Bristol	97
UK	Dundee	50

		
**These centres were divided into regional groups as shown below (see text)**		

Region	Patients in cohort (1520)	Patients in analysis (1327)

Austria and Switzerland	275	242
Germany	575	524
Hungary/Poland	120	97
Spain/Italy/France	165	113
Sweden/Iceland/Finland	238	208
UK	147	143

Scrutiny of the data showed that 193 cases needed to be omitted because of protocol violations. The most common violation was the collection of some of the 'baseline' patient-related information post-operatively (182) and in a further 11 cases it was unclear which hip had been operated on and at a EUROHIP group meeting in 2006 it was agreed that these cases should be omitted from the analysis. The demographic data analysed is therefore on a total of 1327 cases (87%). Unless otherwise stated, all data presented here refer to these 1327 cases.

In order to explore the data for differences within Europe, the participating centres were grouped into 5 regions, (Table 1), providing sufficient numbers in each group to undertake statistical analyses.

While the age range of the included patients was wide, the majority were in their 6^th ^or 7^th ^decade (Table 2). There were more women than men and the group was overweight. Only 25% were still employed at the time of surgery, the majority had retired because of age, but 8% reported that they had retired early because of their hip problems. The patients were generally fairly fit, the ASA status being recorded as grade 1 or 2 in 79%; only 1% of patients were ASA status 4.

**Table 2:** Demographic data

	Patients in analysis (1327)	Excluding missing values
Age groups:		
< 35	6 (0.5%)	6 (0.5%)
35–44	46 (3.5%)	46 (3.5%)
45–54	154 (11.6%)	154 (11.9%)
55–64	373 (28.1%)	373 (28.7%)
65–74	456 (34.4%)	456 (35.1%)
75–84	245 (18.5%)	245 (18.9%)
85+	18 (1.4%)	18 (1.4%)
Missing	29 (2.2%)	
Age (mean, SD)	65.7, 10.9	
Sex:		
Male	559 (42.1%)	559 (44.1%)
Female	708 (53.4%)	708 (55.9%)
Missing	60 (4.5%)	
BMI groups:		
Underweight (< 18.5)	2 (0.2%)	2 (0.2%)
Normal (18.5 to < 25)	359 (27.1%)	359 (29.3%)
Overweight (25 to < 30)	568 (42.8%)	568 (46.4%)
Obese (30 to < 40)	281 (21.2%)	281 (22.9%)
Morbidly Obese (40+)	15 (1.1%)	15 (1.2%)
Missing	102 (7.7%)	
BMI (mean, SD)	27.5, 4.4	
Employment status:		
Employed	325 (24.5%)	325 (25.1%)
Retired	740 (55.8%)	740 (57.2%)
Unemployed	31 (2.3%)	31 (2.4%)
Retired Early	105 (7.9%)	105 (8.1%)
Voluntary work	5 (0.4%)	5 (0.4%)
Housework	88 (6.6%)	88 (6.8%)
Missing	33 (2.5%)	
Qualifications after leaving school:		
Postgraduate degree	52 (3.9%)	52 (4.5%)
University degree	149 (11.2%)	149 (12.9%)
College diploma, or equivalent	371 (28.0%)	371 (32.2%)
None	581 (43.8%)	581 (50.4%)
Missing	174 (13.1%)	
ASA status:		
1	209 (15.8%)	209 (17.8%)
2	719 (54.2%)	719 (61.2%)
3	237 (17.9%)	237 (20.2%)
4	10 (0.8%)	10 (0.9%)
Missing	152 (11.5%)	
Caring for someone else:		
Yes	234 (17.6%)	234 (18.1%)
No	1061 (80.0%)	1061 (81.9%)
Missing	32 (2.4%)	

### 2. Joint disease and joint replacement

As shown in Table 3, the duration of hip pain was generally recorded as 1–5 years; only 30% reported that they had had their hip problem for 6 years or more. Unilateral hip disease was reported by 32%, a further 13% reported bilateral hip disease without involvement of other joints, but the majority (54.6%) had involvement of other joints, and many had undergone previous surgery on other joints (mostly the other hip or the knees). The right hip was operated on more often than the left, and the cemented type of prosthesis was used most commonly.

**Table 3:** Status of hip and other joint disease, and type of prosthesis used

	Patients in analysis (1327)	Excluding missing values
Years of reported pain in the hip to be replaced:		
< 1	141 (10.6%)	141 (10.7%)
1–2	374 (28.2%)	374 (28.5%)
3–5	403 (30.4%)	403 (30.7%)
6–8	147 (11.1%)	147 (11.2%)
9–12	95 (7.2%)	95 (7.2%)
13–15	49 (3.7%)	49 (3.7%)
> 15	105 (7.9%)	105 (8.0%)
Missing	13 (1.0%)	
Arthritis in other joints:		
One hip only	416 (31.4%)	416 (32.4%)
Both hips only	167 (12.6%)	167 (13.0%)
Hips(s) & other peripheral joints	701 (52.8%)	701 (54.6%)
Missing	43 (3.2%)	
Hip joint being replaced:		
Right	724 (54.6%)	724 (54.6%)
Left	603 (45.4%)	603 (45.4%)
Type of prosthesis used:		
Hybrid	246 (18.5%)	246 (19.2%)
Cemented	542 (40.8%)	542 (42.3%)
Uncemented	493 (37.2%)	493 (38.5%)
Missing	46 (3.5%)	
Surgery on other joints in the past:		
Yes	595 (44.8%)	595 (45.4%)
No	716 (54.0%)	716 (54.6%)
Missing	16 (1.2%)	

### 3. Radiographic findings

The inter-rater reliability scores (kappa statistic) for the K&L grades ranged from 0.43 to 0.68, indicating a high degree of agreement between observers. Intra-rater kappa scores ranged from 0.63 to 0.89. Of the 1327 people included in the analyses, radiographs of the operated hip were only available for reading in 1107 cases (610 right side and 497 left), and the readers only felt able to assign a reliable K&L score to 1051 films, because of technical problems or the severity of pathological changes.

**Figure 1:**
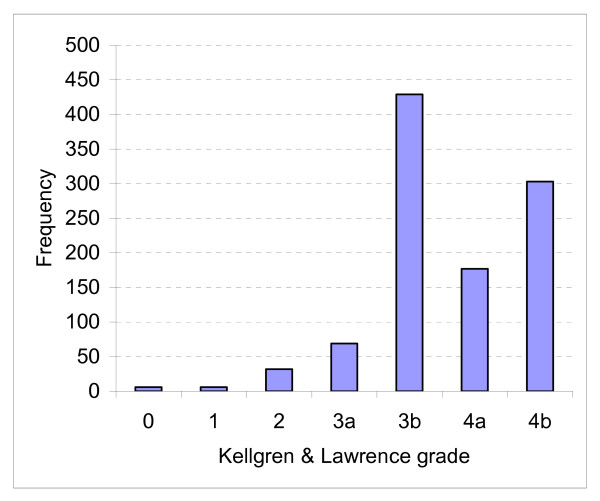
**K&L radiographic scores with the split into 3a and b and 4a and b as described**.

Results for the conventional K&L scoring were grade 0 or 1 – 12 cases (1%), grade 2 – 32 (2.4%), grade 3 – 510 (38.4%), grade 4 – 497 (37.5%), (missing data in 276 cases). We differentiated those with K&L grade 3 or 4 further, as described in the methods section. Of the 510 with grade 3 OA, only 69 had mild joint space narrowing, the remainder having extensive loss of joint space. Of the 497 with grade 4 OA (complete loss of joint space), 303 also had evidence of bone attrition in either the acetabulum or femoral head (Figure 1).

The commonest recorded pattern of the intra-articular pattern of distribution of the OA radiographic changes was superolateral (44.3%), the other patterns, in order of frequency being superomedial (23.5%), medial (19%) and concentric (9.1%). 62 hips (5.9%) were thought to show hypertrophic changes and 102 (9.8%) atrophic features.

### 4. WOMAC scores

**Figure 2:**
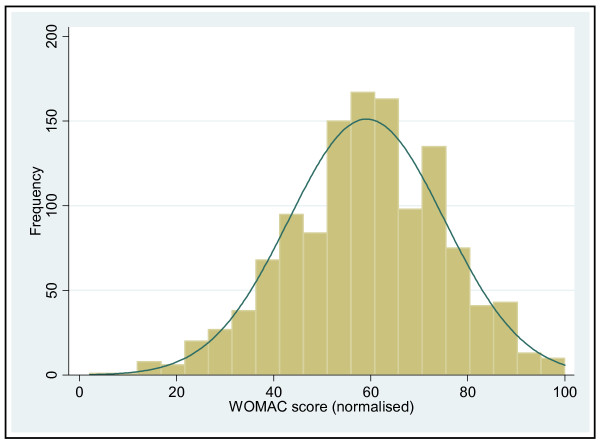
**Histogram of distribution of total WOMAC score**.

Total WOMAC scores were available in 94% of the 1327 patients included in this analysis and followed a normal distribution (Table 4, Figure 2). While the majority of the patients coming to hip replacement had WOMAC scores of 40 or more, a number of patients had relatively low total scores, indicating quite mild pain and disability. Overall pain scores were lower than the stiffness or function domain scores.

**Table 4:** Summary of WOMAC scores

	Pain (n = 1255)	Stiffness (n = 1266)	Function (n = 1253)	Total WOMAC (n = 1243)
Mean (SD)	55.4 (17.8)	60.5 (20.7)	60.1 (16.7)	59.2 (16.1)
Median (IQR)	55 IQR (45–65)	63 IQR (50–75)	60 IQR (50–72)	59.4 IQR (49.0–70.8)

Legend: SD = standard deviation; IQR = interquartile range.

### 5. Associations between demographic features and severity of joint disease

We looked for associations between the total WOMAC scores and age, sex, handedness, BMI, occupational and educational status, ASA status and the K&L score (Table 5). We carried out the same analysis for the WOMAC pain score domain alone, and obtained very similar results (data not shown). It is apparent that higher scores (worse disease) were present in older subjects, women, those with obesity, those with higher ASA status, those who had retired early, and most strikingly, those with no educational qualifications after leaving school. Radiographic scores showed no correlation with WOMAC scores.

**Table 5:** Results of linear regression analysis for Total WOMAC score

	Univariate	Multivariate
	Mean WOMAC score (95%CI)	Mean WOMAC score (95%CI)
Age groups:		
< 50	56.8 (53.8, 59.8)	58.4 (54.7, 62.1)
50–69	58.2 (57.0, 59.4)	57.5 (56.0, 59.0)
70+ (reference)	61.0 (59.5, 62.5)	61.0 (59.2, 62.8)
P for trend	0.002	0.18
P for nonlinear trend	0.51	0.04
Sex:		
Male (reference)	56.1 (54.7, 57.4)	55.7 (54.1, 57.3)
Female	61.5 (60.3, 62.7)	61.6 (60.1, 63.1)
Obesity:		
Not obese (reference)	58.2 (57.1, 59.2)	57.5 (56.2, 58.8)
Obese	62.9 (61.0, 64.8)	62.5 (60.2, 64.7)
Morbidly Obese	69.6 (61.0, 78.1)	67.2 (57.6, 76.7)
P for trend	< 0.001	0.028
P for nonlinear trend	0.68	0.81
Employment status:		
Employed	55.9 (54.1, 57.7)	56.7 (54.6, 58.9)
Retired (reference)	59.6 (58.4, 60.8)	58.8 (57.3, 60.2)
Retired Early	65.9 (62.8, 69.0)	65.3 (61.5, 69.1)
Other	59.8 (56.9, 62.8)	59.6 (55.9, 63.3)
Qualifications after leaving school:		
Postgraduate degree (reference)	49.3 (44.9, 53.7)	48.9 (43.3, 54.6)
University degree	54.0 (51.4, 56.6)	53.8 (50.8, 56.7)
College diploma, or equivalent	56.4 (54.8, 58.1)	56.9 (54.9, 58.9)
None	62.1 (60.8, 63.4)	62.2 (60.7, 63.8)
P for trend	< 0.001	< 0.001
P for nonlinear trend	0.31	0.96
ASA status:		
1	55.5 (53.3, 57.7)	53.2 (50.5, 55.9)
2 (reference)	58.7 (57.5, 59.9)	58.4 (57.0, 59.8)
3	65.6 (63.4, 67.7)	64.6 (62.2, 67.1)
4	59.3 (48.3, 70.3)	54.7 (40.3, 69.0)
P for trend	< 0.001	< 0.001
P for nonlinear trend	0.042	0.06
Kellgren & Lawrence Grade:		
0	60.1 (47.4, 72.8)	53.6 (37.0, 70.1)
1	70.7 (56.7, 84.6)	77.1 (60.6, 93.7)
2	55.7 (50.2, 61.2)	55.4 (49.4, 61.4)
3	58.0 (56.5, 59.4)	58.4 (56.8, 60.0)
4 (reference)	59.9 (58.5, 61.4)	59.4 (57.8, 61.0)
P for trend	0.16	0.66
P for nonlinear trend	0.12	0.14

The square of the correlation coefficient (R-squared) in the multivariate model is 0.164, so the variables included in the regression model account for 16.4% of the variation in the total WOMAC score

We then looked specifically at those with low pain scores (20 or less) in relation to their age and K&L radiographic status (Table 6)

**Table 6:** Number of people with low pain scores by age and K&L grade

	WOMAC Pain score	
	0–20	21–100
Total:	46 (3.7%)	1209 (96.3%)
Age groups:		
< 50	2 (1.8%)	108 (98.2%)
50–69	31 (4.6%)	641 (95.4%)
70+	13 (2.9%)	435 (97.1%)
Kellgren & Lawrence Grade:		
0	0 (0.0%)	6 (100.0%)
1	0 (0.0%)	6 (100.0%)
2	2 (6.3%)	30 (93.8%)
3	15 (3.1%)	467 (96.9%)
4	21 (4.5%)	451 (95.6%)

Finally, we examined the WOMAC total score data to see if there were any obvious differences in scores in the 5 different European regions (Table 7). There were wide variations in each European region, with the trends towards the highest scores in the Eastern Europe grouping (Hungary and Poland) and lowest scores in Austria/Switzerland and the UK. However, there was no evidence to suggest that patients from any one particular region or centre were very different from those of the whole group presented above (data not presented).

**Table 7:** Summary of total WOMAC scores by region (Not been adjusted for potential confounders)

Region:	Mean (SD)	Median (IQR)	Range
Austria and Switzerland	54.1 (17.2)	55.7 IQR (42.7 – 64.6)	11.5 – 97.9
Germany	58.9 (16.2)	59.4 IQR (47.9 – 70.8)	2.1 – 100.0
Hungary/Poland	67.8 (14.8)	66.7 IQR (58.3 – 76.0)	16.7 – 100.0
Spain/Italy/France	61.5 (14.4)	60.4 IQR (52.2 – 71.9)	12.5 – 93.8
Sweden/Iceland/Finland	60.7 (16.3)	61.5 IQR (51.0 – 71.9)	16.7 – 99.0
UK	56.6 (12.1)	58.3 IQR (48.4 – 65.1)	26.0 – 75.0

Legend: SD, standard deviation; IQR, interquartile range.

## Discussion

This large cohort study of patients with hip joint OA coming to primary total hip joint replacement surgery in European orthopaedic centres, shows that disease severity varies greatly at the time of surgery, indicating that there is no consensus on how 'bad' the patient's diseases should be to warrant surgery. The variation was much wider for symptoms than for radiographic changes, and one of the striking findings from the cohort is the absence of any relationship between the two. Other key findings are that the severity of symptoms at the time of surgery is associated with differences in age, sex, weight, general health, employment status and educational status. These variations appear to be present throughout Europe, and not dependent on individual centres.

The demography of our cohort was as expected. The average age of 69, predominance of retired people, greater numbers of women than men, and slightly raised mean BMI are all in accord with other groups of patients coming to hip replacement [21,22]. Similarly, the wide age ranges operated on is in keeping with other data suggesting a trend to surgery being undertaken on more older people [3]. The majority of our cohort had disease of other joints (predominantly the other hip and the knees) but that they were otherwise relatively fit: very few had an ASA score of 3 or 4. The location of the hip OA is also in accord with previous descriptions, with a predominance of supero-lateral or supero-medial disease [23]. One feature of the cohort that did surprise us was the relatively short history of pain in the joint to be operated on: 70% of those for whom we have this information reported 5 years or less of joint pain, and only 12% said that they had suffered for 12 years or more. This suggests that the majority of cases coming to surgery progress fairly rapidly.

We used standard, validated instruments to assess disease severity. The WOMAC is one of the most commonly used disease specific measures, which assesses pain, stiffness and function [16]. One of its problems is the fact that the disability domain, which dominates the total score, cannot differentiate between disability due to a single joint, or that caused by disease in other joints (common in our cohort) or co-morbidities. It has been suggested that people should have a total WOMAC score of 39 or more to be considered for joint replacement [24], but 155 patients (12.5%) of our cohort had a WOMAC score of 40 or less, and 16 patients (1.3%) had total WOMAC scores of 20 or less, which would generally be considered to be indicative of very mild disease. The variation in WOMAC scores shown in Figure 2 is striking. The K&L score is the oldest and most widely used index of radiographic severity of OA [19]. One of its many problems is lack of discrimination and a 'ceiling effect', as established OA can only be scored grade 3 or 4 [25]. We attempted to get round this problem by modifying it so that we have 4 grades of severity of established disease – 3a, 3b, 4a, and 4b. In contrast to the wide variation in the clinical severity of the disease in our cohort, it is clear that the vast majority had severe radiographic changes. This raises concerns that the radiographic findings may be having an undue influence on surgical decision-making. It is not clear if radiographic severity has an impact on the postoperative outcome, and if so, which radiographic features are most predictive, there being some conflicting findings amongst recent publications on this topic [26-28]. Follow-up of our cohort should contribute to this debate. In this context it is again interesting to note the lack of any significant relationship between the clinical severity on the WOMAC score and the radiographic severity of the disease. It is well known from epidemiological studies that while radiographic OA is a clear risk factor for symptoms, the correlation between x-ray changes and symptoms is weak, and that some people with severe x-ray changes of OA remain asymptomatic 29,30. However, the relationship between x-ray changes and symptoms in people with severe arthritis coming to surgery has not been studied extensively, and doctors find it hard to believe that there is not some relationship when the disease is severe. We believe that our finding of no association between x-rays and symptoms in this patient group reinforces the need to assess patients on the basis of the impact of the condition on their lives, and not on x-ray severity, when considering them for surgery.

Some of our data on the determinants of the variation in the severity of pain and disability at the time of surgery are worrying. Similar sex differences have been found in several other studies of surgical interventions (women always having more severe disease at the time of surgery than men), and remain largely unexplained [24]. The fact that those who were more unfit (higher ASA status) or more obese, had more severe symptoms than average before they were operated on, is also neither a new nor a surprising finding. However, the strong association with both employment status and educational status are new findings. There are several possible explanations for an association between socio-economic status and surgery, including access and willingness to undergo surgery, as well as bias amongst health care professionals, we believe this inequality needs further investigation.

Our initial exploration of the data to find out if the variations in severity observed, and their determinants, are dominated by events in a single region of Europe, or single centres (data not shown) suggests that these findings are fairly consistent across all countries and centres studied. However, further examination of the data centre by centre is being undertaken. The multi-centre, multi-country nature of the cohort is both a strength and a weakness. The strength is that it will allow us to examine differences in different parts of Europe, the weakness is that the huge variations in the health care systems between, for example, northern and eastern Europe, introduce another source of variation that has nothing to do with the patients' need for surgery.

This cohort study has other strengths and limitations. Its strengths include the relatively large number of patients involved, use of validated outcome measures, and relative paucity of missing data. Limitations include the fact that we do not know how representative the cohort is, as it came from self-selected centres rather than a random selection of orthopaedic centres, and the fact that we do not know how many patients were excluded from the study in each centre, or the reasons for exclusion. In addition, we do not know which potential patients might have been triaged out of the system before being able to see a surgeon, or how many were not put on the waiting list for surgery. In other words, we do not know whether the patients in our cohort are truly representative of those in need of a THR in the community. It is known that the willingness of patients to undergo surgery determines the utilisation of THR [31], and it is likely that this varies in different European countries.

There are no clear indications for THR. Consensus statements simply emphasise pain due to hip disease and functional impairment, in spite of adequate non-surgical treatment [4,9]. However, clinical practitioners are aware of various other reasons for undertaking or delaying a hip replacement, including age and weight, the social role of the patient (as a carer for example), their psychological status, and the presence or absence of any co-morbidities that might affect surgery or its outcome. In addition, surgeons are aware of the importance of the motivation and expectations of their patients when they undertake surgery. There is an increasing trend for those who pay for surgical interventions, such as total hip replacement, to want to use simple scoring systems in order to 'triage' patients as suitable or unsuitable for surgery. But simple scoring systems, such as the total WOMAC, cannot account for subtleties of the sort noted, and these are crucial to appropriate decision making. It has recently been suggested that a different approach, using the concept of the 'capacity to benefit' from a total joint replacement might be used instead of simple scoring systems [7]. This approach provides surgeons and patients with information on the likely risk of adverse events, as well as the likely degree of benefit, from which to make the judgment on suitability of surgery. Our data show clearly that if a score of severity of pain or function were used to assess suitability for surgery, many patients who are currently being operated on might not be allowed an operation. One of the aims of the 'EUROHIP' consortium is to explore this conundrum further, with more exploration of these and other data to help us understand when it is most appropriate to undertake a hip replacement.

## Conclusion

This large prospective cohort study of patients coming to primary total hip replacement for primary osteoarthritis of the hip has shown that the severity of the disease varies widely at the time of surgery. The pre-operative scores on the WOMAC instrument, a widely used measure of osteoarthritis severity that assesses pain, stiffness and function, show that many of the patients appeared to have relatively mild disease, whereas others are very severely affected. In contrast, the radiographic findings showed that almost all patients coming to surgery had severe structural changes in the affected hip. There was no correlation between clinical severity and radiographic severity.

We interpret these findings as follows. First, we believe that the data indicate that surgeons require significant structural changes to be present on the radiographs of their patients before they are willing to suggest that hip replacement is advisable. Second, these data show that simple scoring systems of pain and disability, such as the WOMAC, should not be used to define thresholds for surgical intervention. The lack of correlation between radiographic severity and clinical severity on the WOMAC suggests that the decision needs to be based on patient-related variables rather than the x-ray, and those variables should include the degree of pain and disability. However, a large number of other aspects of the patients' lives need to be considered before surgery is undertaken, including their psychological status, their motivation and expectations, roles in society and social circumstances. We believe that the patients enrolled into this cohort study were being operated on for these complex reasons, and that this explains the huge variation seen in pre-operative WOMAC scores.

## Competing interests

The authors declare that they have no competing interests.

## Authors' contributions

The principle investigators of the EUROHIP cohort study are PD and KD. The data collection was undertaken by all contributing centres and co-ordinated by SW and MF in collaboration with PD and KD. Statistical analysis was carried out by AJ and II. The manuscript was drafted by PD. All members of this EUROHIP study group advised and/or contributed to the design and data collection for this study. All authors made significant contributions to the interpretation of the data and the final manuscript.

## Pre-publication history

The pre-publication history for this paper can be accessed here:


